# Exploring Pathogenic Genes in Frozen Shoulder through weighted gene co-expression network analysis and Mendelian Randomization

**DOI:** 10.7150/ijms.98505

**Published:** 2024-10-21

**Authors:** Dusu Wen, Bin Li, Shun Guo, Liaobin Chen, Biao Chen

**Affiliations:** Division of Joint Surgery and Sports Medicine, Department of Orthopedic Surgery, Zhongnan Hospital of Wuhan University, Wuhan 430071, China.

**Keywords:** frozen shoulder, differentially expressed genes, weighted gene co-expression network analysis, mendelian randomization, immune cell infiltration

## Abstract

**Background:** Frozen shoulder (FS) is characterized by the thickening and fibrosis of the joint capsule, leading to joint contracture and a reduction in joint volume. The precise etiology responsible for these pathological changes remains elusive. Therefore, the primary aim of this study was to explore the potential involvement of pathogenic genes in FS and analyze their underlying roles in the disease progression.

**Methods:** Differential expression analysis and weighted gene co-expression network analysis (WGCNA) were employed to investigate co-expressed genes potentially associated with FS. Gene Ontology and Kyoto Encyclopedia of Genes and Genomes analyses were conducted to elucidate the potential roles of these co-expressed genes. Subsequently, Mendelian randomization (MR) analysis was performed using expression quantitative trait loci datasets for the co-expressed genes, combined with summary statistics from the genome-wide association study of FS, aiming to identify key genes causally associated with FS. The identified key genes were further validated through reverse transcription-quantitative PCR (RT-qPCR). Additionally, a nomogram model and receiver operating characteristic (ROC) curves were established to assess the diagnostic value of the hub genes. Furthermore, the infiltration of immune cells was evaluated using the CIBERSORT algorithm and the relationship between key genes and immune-infiltrating cells was analyzed.

**Results:** 295 overlapping co-expressed genes were identified by intersecting the differentially expressed genes with the hub genes obtained from associated modules identified through WGCNA. Utilizing MR analysis, four key genes, namely ADAMTS1, NR4A2, PARD6G and SMKR1, were found to exhibit positive causal relationships with FS, which were subsequently validated through RT-qPCR analysis. Moreover, the diagnostic value of these four key genes was demonstrated through the development of a nomogram model and the construction of ROC curves. Notably, a causal relationship between ADAMTS1 and immune cell infiltration in FS was observed.

**Conclusion:** Our study suggested genetic predisposition to higher expression levels of ADAMTS1, NR4A2, PARD6G and SMKR1, was associated with an increased risk of FS. Further investigations elucidating the functional roles of these genes will enhance our understanding of the pathogenesis of FS and may facilitate the development of targeted treatment strategies.

## 1. Introduction

Frozen shoulder (FS), also referred to as adhesive capsulitis, is a prevalent shoulder disorder characterized by a gradual reduction in glenohumeral joint mobility accompanied by pain 1. Pathologically, it disrupts typical collagen structures, leading to progressive fibrosis of connective tissues and thickening of the adjacent synovium. These fibrotic processes are accompanied by inflammation, neovascularization, and new neural innervation 2, 3, resulting in a decrease in joint volume and an increase in the stiffness of the joint capsule. Although FS is self-limiting and often resolves spontaneously within 1-2 years, studies indicate that 20%-50% of patients may continue to experience symptoms, including stiffness and pain 4, 5. Prioritizing preventive measures is crucial for alleviating patient symptoms and reducing the socio-medical burden. Therefore, identifying new risk factors and interventions for FS through further research into its pathophysiology is imperative, aiming to discover reliable biomarkers for early detection and treatment of the condition.

Identifying the expression of specific genes is crucial for comprehending the underlying micro-mechanisms of FS and discerning relevant biomarkers for disease diagnosis and treatment assessment. Weighted gene co-expression network analysis (WGCNA) stands as an unbiased systematic biological analysis method crafted to identify co-expressed gene modules, investigate the correlation between gene networks and phenotypes of interest, and pinpoint hub genes within these networks 6. Furthermore, WGCNA possesses the capacity to screen for potential therapeutic targets or candidate biomarkers. However, the application of the WGCNA method for bioinformatics analysis in FS remains largely unexplored. Hence, this study aims to employ WGCNA to uncover hub genes, identify new biomarkers, and elucidate potential pathological mechanisms associated with FS.

Mendelian randomization (MR) is a powerful tool for investigating potential causal relationships between exposures and outcomes by utilizing single nucleotide polymorphisms (SNPs) as instrumental variables (IVs) for the exposure 7. Compared to traditional statistical methods used in association studies, MR effectively mitigates confounding factors and reverse causation, making it an increasingly popular approach for exploring etiological mechanisms 8, 9. Expression quantitative trait loci (eQTLs) are genetic loci that influence gene expression levels, with many being SNPs. Specific SNPs associated with changes in gene expression play a crucial role in transcriptomics by facilitating the identification of genetic markers 10, 11. These identified gene markers can reflect an individual's health status, providing novel insights into the underlying mechanisms of various diseases, including cardiovascular diseases, systemic lupus erythematosus, depression, inflammatory bowel disease, and osteoporosis 12-17. This underscores the potential of eQTLs as valuable tools for investigating genes that are pleiotropically associated with complex traits. By employing MR analysis to assess the association between the eQTLs of identified key genes and the risk of FS, we can determine whether the expression of candidate genes selected through WGCNA has a causal effect on FS risk. This approach facilitates the discovery of novel hub genes and provides clarity regarding the control mechanisms underlying the pathogenesis of FS, ultimately aiding in the identification of new therapeutic targets for its treatment.

In this study, we utilized the GSE140731 RNA-seq dataset from the Gene Expression Omnibus (GEO) database to identify co-expressed genes by intersecting the differentially expressed genes (DEGs) with hub genes obtained from key modules identified through WGCNA. Subsequently, we conducted a MR study to examine the causal effects of eQTLs, derived from the co-expressed genes, on the risk of FS using data from genome-wide association studies. To validate our findings, we further analyzed the expression of these genes in clinical samples. The diagnostic value of the identified key genes was evaluated using nomogram models and Receiver Operating Characteristic (ROC) analysis. Finally, we employed CIBERSORT software to analyze discrepancies in immune cell infiltration and to explore the interactions between the key genes and infiltrating immune cells.

## 2. Materials and methods

### 2.1 Downloading and Processing of Data

Initially, we downloaded the GSE140731 transcriptome data from the GEO database 18. The GSE140731 database contained 22 control samples and 26 FS samples. Subsequently, the transcriptome data were transformed to log2 values and quantile normalized using the normalizeBetweenArrays function from the "limma" package in R (version 4.2.2; https://www.r-project.org/) 19. Differentially expressed genes (DEGs) were identified based on an adjusted p-value (adj.P.Val) < 0.05 and |log fold change (logFC)| ≥ 1. The "ggplot2" package in R was employed to create volcano plots and heatmaps for the statistical analysis of expression levels.

### 2.2 WGCNA

WGCNA is a method utilized to construct gene co-expression networks and to investigate the relationships between phenotypes and gene expression levels. In this study, we employed the "WGCNA" package to construct a co-expression network 6 and identify significant modules using the FS transcriptome data dataset, GSE140731. Briefly, the weighted adjacency matrix was constructed using the soft-thresholding power (β) of 10 to attain scale-free topology. This adjacency matrix was then transformed into a topologically overlapping matrix and subsequently into a dissimilarity matrix. The dissimilarity matrix was used to perform hierarchical clustering of the genes, which were assigned to different modules. Modules were identified using dynamic tree cutting, with a minimum module size set at 50 genes. To quantify the co-expression similarity of the entire modules, we calculated their eigengenes and clustered them based on correlation. A correlation of 75% (distance threshold of 0.25) was used to merge similar modules. The module eigengenes were then correlated with FS, and associations with a p-value of less than 0.05 were considered statistically significant. Genes corresponding to significantly correlated modules and exhibiting significant differential expression between FS and control samples were selected for further analysis.

### 2.3 Screening of co-expressed genes and GO/KEGG analysis

Genes identified from the intersection of DEGs and WGCNA analysis were considered relevant co-expressed genes associated with fibromyalgia syndrome (FS). To further investigate the functional roles of these co-expressed genes, functional enrichment analysis was performed. Gene Ontology (GO) provides a widely used framework for annotating genes based on their functions, categorizing them into three main aspects: molecular function (MF), biological process (BP), and cellular component (CC). Additionally, Kyoto Encyclopedia of Genes and Genomes (KEGG) enrichment analysis serves as a valuable resource for studying gene functions and understanding high-level genomic information 20. To elucidate the involvement of co-expressed in the pathogenesis of FS, we utilized the "ClusterProfiler" package in R to analyze the GO functions of these genes and their enrichment in KEGG pathways. Heatmaps were generated using the "pheatmap" package in R. In this analysis, the minimum gene set size was set to 1, and the maximum was capped at 5,000. A p-value of less than 0.05 was considered indicative of significantly enriched terms.

### 2.4 MR analysis

MR analysis employs genome-wide significant SNPs as IVs to investigate the causal effects of a specific exposure on an outcome. This analytical approach has been extensively utilized to elucidate the genetic etiology of complex diseases by integrating quantitative trait loci data 21. The eQTLs data were obtained from genome-wide association study (GWAS) data (https://gwas.mrcieu.ac.uk/). To select eQTLs, we applied the following criteria: (1) eQTLs exhibited genome-wide significant associations (p < 5E-08), (2) the assumption of independence was met by ensuring linkage disequilibrium (LD) clumping with an r² threshold of less than 0.001, and (3) eQTLs were not considered weak IVs, having F-statistics greater than 10. In total, SNPs for 510 genes were included in the analysis. GWAS data specific to FS were obtained from the IEU OpenGWAS project (https://gwas.mrcieu.ac.uk/) 22. All data utilized in this study were derived from European populations, including data for FS (ebi-a-GCST90000512), which included 15,184,371 SNPs with a total sample size of 451,099. This study reanalyzed previously collected and publicly available data and therefore did not require additional ethical approval.

The selection of appropriate IVs is crucial for MR analysis, serving as the initial and paramount step in conducting this methodology. To satisfy Assumption 1 (relevance assumption), SNPs must exhibit a strong association with the exposure of interest. In this study, we applied a genome-wide significance threshold of p < 5E-08 to filter the SNPs obtained from the eQTLs dataset as IVs. Assumption 2 (independence assumption) necessitates that the IVs are not associated with confounding factors, which can be assessed through the examination of pleiotropy in post-MR analysis. Lastly, Assumption 3 (exclusion assumption) mandates that the IVs do not have a direct association with the outcome under investigation.

Then, the selected IVs underwent linkage disequilibrium clumping with an r^2^ < 0.001 within a 10-megabase distance. Subsequently, the IVs were harmonized with the outcome variable. Then, according to the number of SNPs corresponding to each gene, different MR analysis strategies were used. The Wald ratio method was utilized when only one SNP was available for one gene, while the Inverse variance weighting (IVW) method, Egger's regression and Weighted median methods were employed for cases with two or more SNPs for another gene 23. An odds ratio (OR) greater than 1 implies a risk factor and OR less than 1 implies a protective factor. Genes meeting the criteria of OR > 1 and logFC>1 or OR < 1 and logFC<-1 criteria were considered to have a positive causal relationship with an increased risk of FS. The genes obtained from the intersecting between the OR and logFC were considered to be hub genes associated with FS. These procedures were implemented using the "TwoSampleMR" R package (github.com/MRCIEU/TwoSampleMR).

### 2.5 Clinical specimen collection

This study received approval from the Ethics Committee of Zhongnan Hospital of Wuhan University (Approval Number: 2022023K). All procedures involving human participants in this study were conducted in accordance with the Helsinki Declaration (2013 revision). Patient Selection: Diagnosis of FS was established through a combination of medical history, physical examination, and arthroscopic findings. Patients with rotator cuff tears and unrestricted passive range of motion (ROM) were selected as the control group. All patients were required to be between 40 and 70 years of age, with FS patients requiring a symptom duration of less than 12 months. Exclusion criteria for the FS group included calcific tendinitis of the shoulder, joint infection, joint instability, rheumatoid arthritis, glenohumeral joint arthritis, and prior shoulder joint surgery. Tissue Collection: Synovial tissue samples were obtained from 12 FS patients and 12 rotator cuff tear patients (Supplementary Table 1) using 3.5 mm biopsy forceps under arthroscopic visualization. The synovial tissue was immediately transferred to RNAlater solution (Thermo Fisher Scientific) post-surgery, refrigerated overnight at 4°C, and stored at -80°C.

### 2.6 Histological analysis

Six random synovial tissue sections were selected from both the FS patient group and the control group. These sections were immediately fixed in 10% formaldehyde for 16-24 hours. Subsequently, the samples were embedded in paraffin, cut into 4-6 μm sections, and underwent hematoxylin and eosin (H&E) staining. Following deparaffinization and dehydration, the sections were stained with hematoxylin for 3-5 minutes, followed by three washes and clearance in 1% hydrochloric acid alcohol. Eosin staining was then performed for 2-3 minutes. The specimens were fixed in 10% formaldehyde, embedded in paraffin, and sagittally sectioned. Masson's trichrome staining was conducted to evaluate the extent of fibrosis.

### 2.7 Reverse transcription-quantitative PCR (RT-qPCR)

Total RNA was extracted from human shoulder joint synovial tissue using Trizol reagent according to the manufacturer's instructions. CDNA was synthesized using the cDNA Synthesis SuperMix Kit. RT-qPCR was performed using the SYBR Green PCR Master Mix and an ABI StepOne instrument (Applied Biosystems, Foster City, CA, USA). Each 20 µL reaction well contained 10 µL of 2× SYBR qPCR Mix, 1 µL of forward primer, 1 µL of reverse primer, 6 µL of RNAse-free water, and 2 µL of cDNA template. The reaction conditions were as follows: 95°C for 10 minutes, followed by 35 cycles of 95°C for 15 seconds, 60°C for 20 seconds, and 72°C for 15 seconds. Relative gene expression levels were calculated using 2^-ΔΔCT^ relative to glyceraldehyde-3-phosphate dehydrogenase (GAPDH). All primers were designed using the Primer 5.0 program (Premier Biosoft International, Palo Alto, CA.) and were synthesized by Wuhan Saiweier Biotechnology Co., Ltd (Wuhan, China). The following is a list of the primer sequences: GAPDH (Forward: 5-GGAAGCTTGTCATCAATGGAAATC-3; Reverse: 5-TGATGACCCTTTTGGCTCCC-3). ADAM metallopeptidase with thrombospondin type 1 motif 1 (ADAMTS1) (Forward: 5-TTGATAAATGTGGTGTTTGCGG-3; Reverse: 5-TCATGATATCCAGGTTTTGCACTAG-3); Nuclear receptor subfamily 4 group A member 2 (NR4A2) (Forward: 5-CAGTGGAGGGTAAACTCATCTTTTG-3; Reverse: 5-CCCGTGTCTCTCTGTGACCATAG-3); Par-6 family cell polarity regulator Gamma (PARD6G) (Forward: 5-CTGGCTGTGAATGACGAGGT-3; Reverse: 5-GACGGTGACGATGAGGTTGT-3); Small lysine rich protein 1 (SMKR1) (Forward: 5-AACCTCTACTACATCGCCCACAAC-3; Reverse: 5-GTCACTTGCTTCTCCCTTTCTTTC-3).

### 2.8 Nomogram model construction

To predict the risk of FS, we developed a nomogram model utilizing the "rms" package. The performance of the nomogram model was evaluated by calculating Harrell's concordance index, which provided an assessment of its predictive power. Additionally, the diagnostic effectiveness of the candidate biomarkers was validated using the "ROC" package to construct a ROC curve. The accuracy of the model was indicated by the area under the ROC curve (AUC). AUC >0.7 was considered acceptable 24.

### 2.9 Immune infiltration analysis

To estimate the relative proportions of immune cells infiltrating the included samples, we utilized the CIBERSORT algorithm, which provides estimates for 22 different immune cell types 25. Samples with a CIBERSORT output of p<0.05 were deemed accurate and included in the construction of the immune landscape, while samples with higher p-values were excluded. Subsequently, we constructed and visualized a correlation matrix of the different immune cell types using the "Corrplot" R package. The analysis of the Spearman relationship between characteristic diagnostic markers and immune infiltrating cells, as well as the visualization of the results, was conducted using the "ggstatsplot" and "ggplot2" packages.

### 2.10 Statistical analysis

Experimental data analysis was conducted using Prism 9.0 software (GraphPad Software, La Jolla, CA, United States). The data were presented as mean values ± standard error of the mean (S.E.M.). The student's two-tailed t-test was employed for group comparisons where applicable. Statistical significance was defined as P < 0.05.

## 3. Results

### 3.1. Identification of DEGs

**Figure 1:**
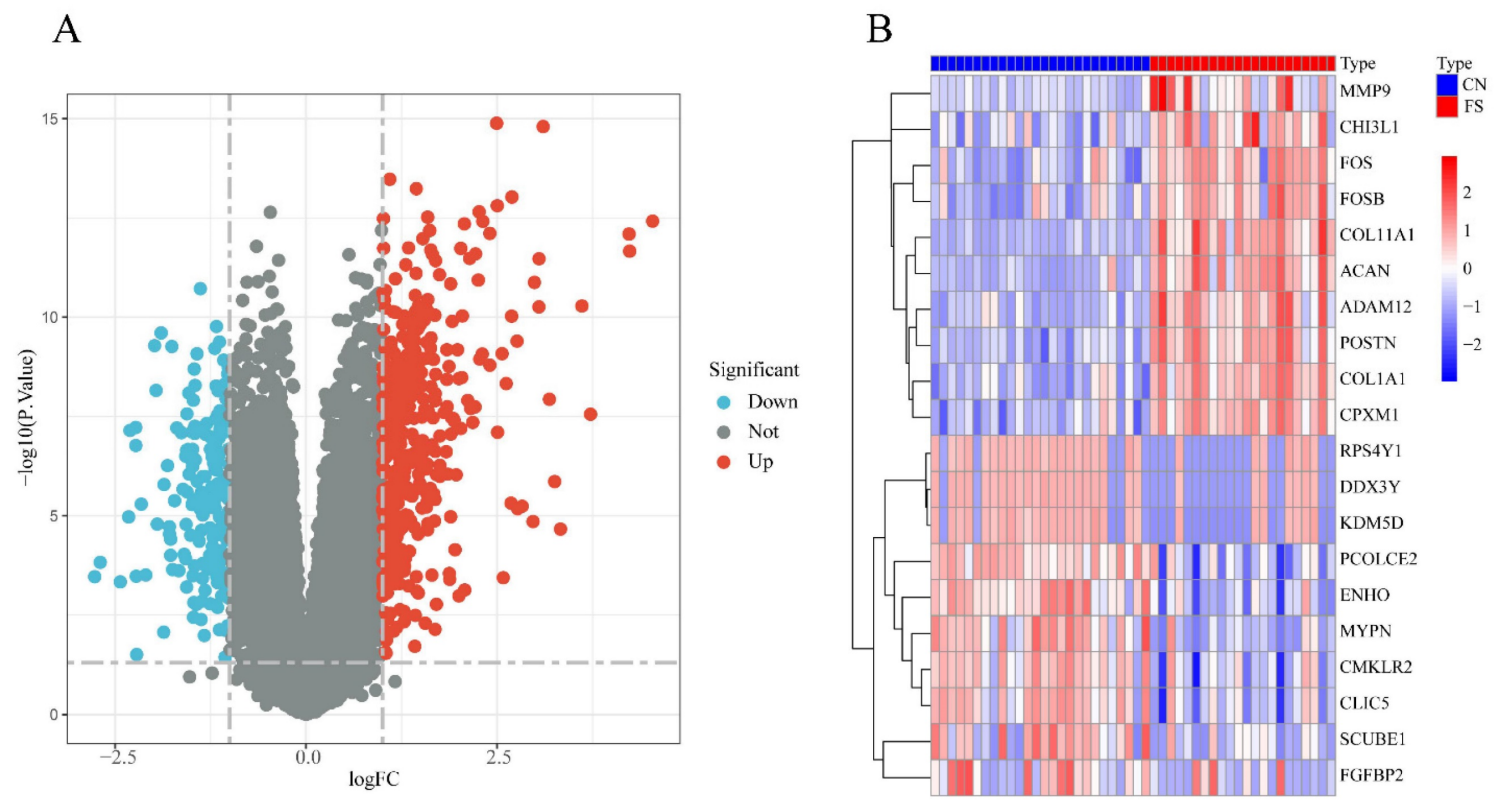
** Differentially expressed genes analysis between FS and control groups in the GSE140731 dataset.** (A) Volcano plot illustrating the differentially expressed genes according to the criteria of an adj.P.Val< 0.05 and |logFC| >1.0. Upregulated genes are represented in red, while downregulated genes are shown in blue. (B) Heatmap depicting the top 10 DEGs identified in the FS group of the GSE140731 dataset.

The FS dataset, GSE140731, was sourced from the GEO database, and the DEGs were determined using the criteria of an adj.P.Val< 0.05 and |logFC | >1. As depicted in Figure 1A, a total of 549 DEGs were identified, including 374 upregulated and 175 downregulated genes in the FS group compared to the control group. Among the upregulated genes, the top 10 based on logFC were: POSTN (logFC = 4.54), CPXM1 (logFC = 4.24), ACAN (logFC = 4.23), FOSB (logFC = 3.72), ADAM12 (logFC = 3.61), MMP9 (logFC = 3.33), CHI3L1 (logFC = 3.25), FOS (logFC = 3.18), COL11A1 (logFC = 3.10), and COL1A1 (logFC = 3.05). Conversely, the top 10 downregulated genes were: RPS4Y1 (logFC = -2.77), DDX3Y (logFC = -2.69), SCUBE1 (logFC = -2.43), CMKLR2 (logFC = -2.32), PCOLCE2 (logFC = -2.30), ENHO (logFC = -2.23), MYPN (logFC = -2.23), KDM5D (logFC = -2.22), CLIC5 (logFC = -2.16), and FGFBP2 (logFC = -2.10) (Figure 1B).

### 3.2. Construction of WGCNA network and identification of FS-related module

**Figure 2:**
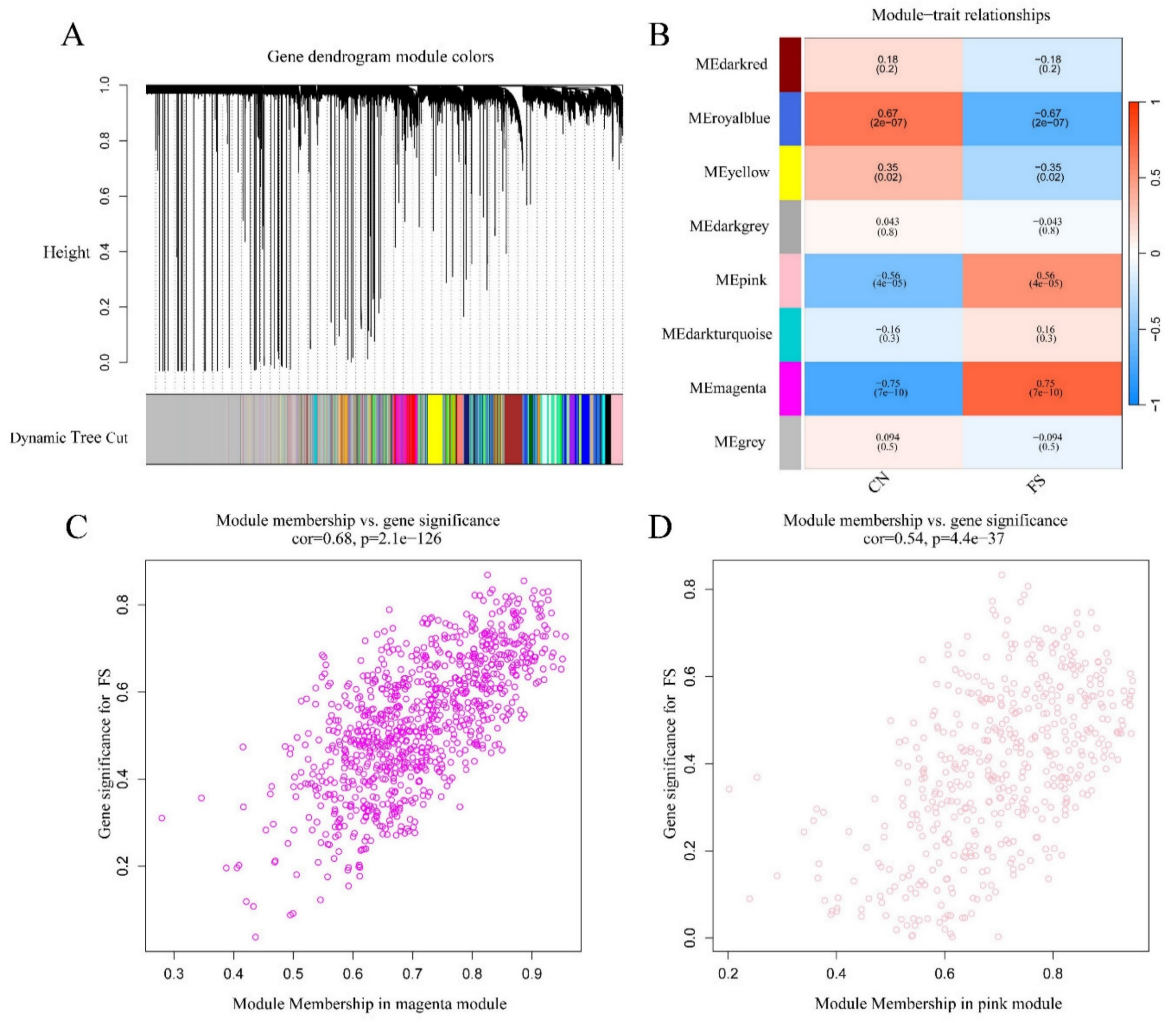
** Identification of gene modules associated with FS using WGCNA.** (A) Clustering dendrogram of genes, with dissimilarity measured based on topological overlap, alongside assigned module colors. A total of 28 co-expression modules were constructed and are represented in different colors as determined by the dynamic tree-cut method. (B) The heatmap displays the correlation and p-values between module eigengenes and FS status. The numbers in the heatmap represent the correlations of the respective module eigengenes with the clinical trait, with p-values shown in parentheses. The intensity of the color indicates the strength of the correlation, with associations having p < 0.05 considered significant. (C) Scatter plot illustrating gene significance for FS versus module membership in the magenta module. (D) Scatter plot illustrating gene significance for FS versus module membership in the pink module.

To investigate the potential association between gene modules and FS, we performed WGCNA using the dataset GSE140731. As illustrated in Figure 2A, a gene dendrogram was generated by clustering genes based on their dissimilarity, which was computed using consensus topological overlap. The colored rows indicate initial module assignments, revealing a total of 28 preliminary modules identified from the clustering of 16,418 genes. To identify modules significantly associated with FS, we summarized the expression profiles of each module as the eigenvector correlated with the first principal component of the expression matrix, referred to as the Module Eigengene (ME) 26. As shown in Figure 2B, the module-trait analysis indicated that the pink, magenta, royal blue, and yellow modules exhibited correlations with the clinical state of FS (cor = 0.56, P = 4e-05; cor = -0.75, P = 7e-10; cor = -0.67, P = 2e-07; cor = -0.35, P = 0.02, respectively). In contrast, other modules, including dark red, dark grey, dark turquoise, and ME grey, were not significantly correlated with FS diagnosis. Due to their positive association with FS, we selected the pink and magenta modules for further analysis.

Values of gene significance (GS) were calculated to assess the association of individual genes with FS. Additionally, Module Membership (MM) was defined as the correlation between the ME and the gene expression profile for each module. When GS and MM were found to be strongly correlated, the most important (central) elements in the modules were closely related to the trait and could be considered hub genes 26. Scatter plots depicting the relationship between MM and GS for each gene in the modules are shown in Figures 2C and 2D. In the magenta module (472 genes), GS was highly correlated with MM (cor = 0.68, p = 2.1e-126) (Figure 2C), while the pink module (924 genes) also exhibited a significant correlation (cor = 0.54, p = 4.4e-37) (Figure 2D). Therefore, a total of 1,396 crucial genes (472 genes from the magenta module plus 924 genes from the pink module) were identified as hub genes significantly associated with FS.

### 3.3 Functional enrichment analysis

**Figure 3:**
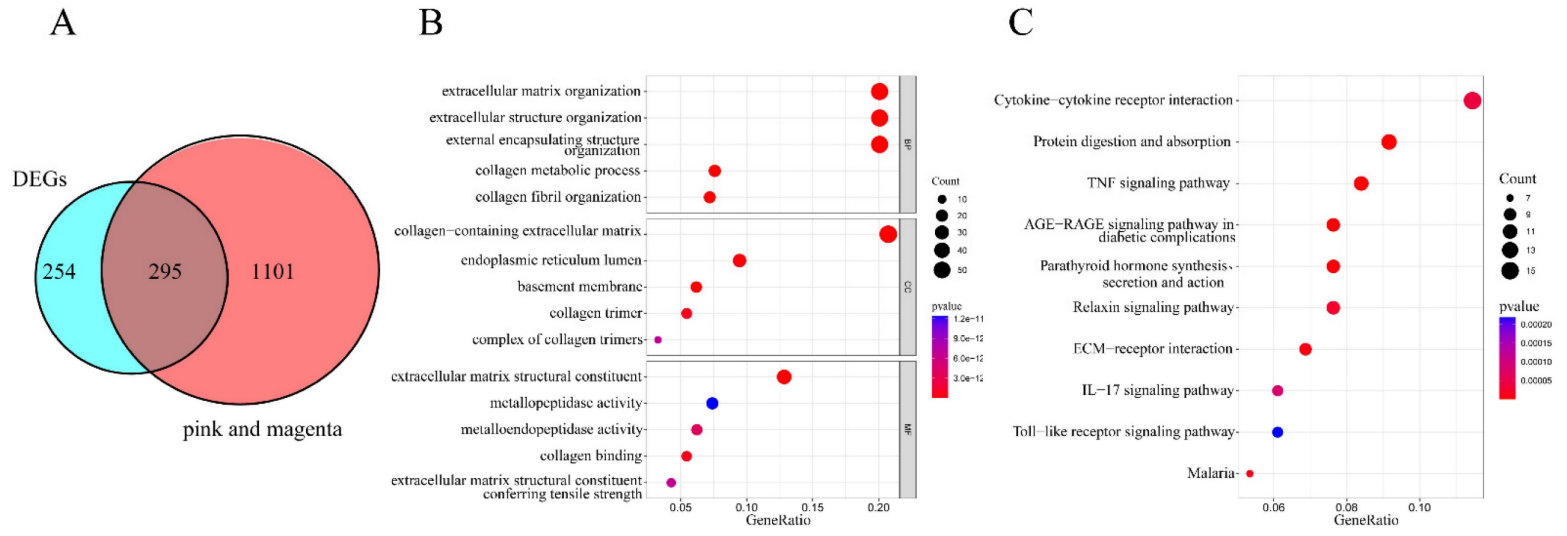
** Functional enrichment analysis of co-expressed genes.** (A) A Venn diagram illustrating the identification of 295 overlapping co-expressed genes. (B) Bubble plot depicting Gene Ontology (GO) enrichment analysis, which includes the top five categories for biological processes (BP), molecular functions (MF), and cellular components (CC). The "Count" indicates the number of co-expressed genes enriched in each pathway, while "GeneRatio" represents the ratio of enriched co-expressed genes to background genes. The X-axis denotes the Gene Ratio, and the Y-axis represents the p-values for the various GO terms. The size of each bubble corresponds to the number of genes enriched for each specific GO term, with redder colors indicating smaller p-values. (C) Bubble plot displaying the top 10 KEGG pathways enriched with co-expressed genes. "Count" indicates the number of co-expressed genes enriched in each pathway, and "GeneRatio" reflects the ratio of enriched genes to background genes. The X-axis represents the Gene Ratio, and the Y-axis shows the p-values for the KEGG pathways. The size of each bubble corresponds to the number of genes enriched for each specific GO term, with redder colors indicating smaller p-values.

To identify co-expressed genes potentially involved in the development and progression of FS, we intersected the 549 DEGs with the 1396 hub genes derived from the magenta and pink modules identified in the WGCNA analysis. As shown in Figure 3A, 295 overlapping genes were identified. Further exploration of these overlapping genes was conducted through GO and KEGG analyses, with all GO terms and KEGG pathways listed in Supplementary Table S2 and S3. The top five categories for GO terms were presented in Figure 3B. In this analysis, the "Count" indicated the number of co-expressed genes enriched in each pathway, while the "GeneRatio" represented the ratio of enriched co-expressed genes to background genes. The size of each bubble corresponds to the number of genes enriched for each specific GO term, with redder colors indicating smaller p-values. Specifically, GO Biological Process (BP) analysis revealed that the co-expressed genes were predominantly enriched in extracellular matrix organization, extracellular structure organization, and external encapsulating structure organization. GO Cellular Component (CC) analysis indicated that these genes were involved in the collagen-containing extracellular matrix, endoplasmic reticulum lumen, and basement membrane. Additionally, GO Molecular Function (MF) analysis highlighted their associations with extracellular matrix structural constituents, metallopeptidase activity, and metalloendopeptidase activity. The top 10 pathways from the KEGG enrichment analysis were presented in Figure 3C. The co-expressed genes were primarily enriched in the following pathways: cytokine-cytokine receptor interaction, protein digestion and absorption, tumor necrosis factor (TNF) signaling pathway, advanced glycation end-product (AGE)-receptor for advanced glycation end-products (RAGE) signaling pathway in diabetic complications, parathyroid hormone synthesis, secretion, and action, relaxin signaling pathway, extracellular matrix-receptor interaction, interleukin-17 (IL-17) signaling pathway, Toll-like receptor signaling pathway, and malaria.

### 3.4 MR analysis

The above analysis indicated that these 295 co-expressed genes may be associated with FS. To evaluate the potential causal relationships between gene expression and FS risk, we first screened eQTLs that affect the expression of the co-expressed genes from the eQTLs gene database. We then utilized SNPs associated with these eQTLs as instrumental variables and performed MR analysis. Specifically, among the 295 co-expressed genes, 111 were excluded due to a lack of suitable SNPs, resulting in 510 SNPs screened for the remaining 184 genes. The selected SNPs exhibited F statistics exceeding 10 (Supplementary Table 4). Depending on the number of SNPs corresponding to each gene, different MR analysis strategies were employed. The Wald ratio method was used when only one SNP was available for a gene, while the inverse variance weighting (IVW) method, Egger's regression, and weighted median methods were employed for genes with two or more SNPs 2. All MR analyses related to the 510 SNPs across 184 genes are presented in Supplementary Table 5.

An odds ratio (OR) greater than 1 indicates a risk factor, while an OR less than 1 indicates a protective factor. Genes meeting the criteria of OR > 1 and logFC > 1, or OR < 1 and logFC < -1, were considered to have a positive causal relationship with an increased risk of FS. Consequently, four genes—NR4A2 (1 SNP), PARD6G (1 SNP), SMKR1 (1 SNP), and ADAMTS1 (4 SNPs)—were identified (Table 1). The results from the Wald ratio analysis demonstrated that genetic liability associated with higher expression levels of NR4A2, PARD6G, and SMKR1 was significantly linked to an increased risk of FS (p = 0.03, p = 0.02, and p = 0.01, respectively). Specifically, for each standard deviation increase in the expression of NR4A2, PARD6G, and SMKR1, the ORs and 95% confidence intervals (CIs) for FS were OR = 1.28 (95% CI: 1.02-1.61), OR = 1.27 (95% CI: 1.04-1.54), and OR = 1.50 (95% CI: 1.12-2.02), respectively.

Regarding ADAMTS1 with 4 SNPs, the primary IVW result indicated that a genetic predisposition to higher expression levels of ADAMTS1 was associated with an increased risk of FS, yielding an OR of 1.12 (95% CI: 1.02-1.24, p = 0.02). For a 1 standard deviation increment in ADAMTS1 expression, the OR was 1.12 (95% CI: 1.02-1.24). This association was consistent across the weighted median method (OR = 1.15, 95% CI: 1.06-1.24, p = 0.01) and the MR-Egger method (OR = 1.22, 95% CI: 1.09-1.36, p = 0.07).

Additionally, MR-Egger regression and IVW analysis were conducted to assess heterogeneity, revealing no significant heterogeneity in the MR analyses of ADAMTS1 for FS (Supplementary Table 6). The MR-Egger intercept tests indicated no evidence of horizontal pleiotropy, as all p-values exceeded 0.05 (Supplementary Table 6). The scatter plot depicted the estimated impact of SNPs on ADAMTS1 expression and FS (Supplementary Figure 1A), while the leave-one-out analysis confirmed that no outlier instrumental variables significantly influenced the overall results (Supplementary Figure 1B). The funnel plot for the ADAMTS1-FS analysis further suggested no apparent horizontal pleiotropy (Supplementary Figure 1C).

### 3.5 Experimental validation

**Figure 4:**
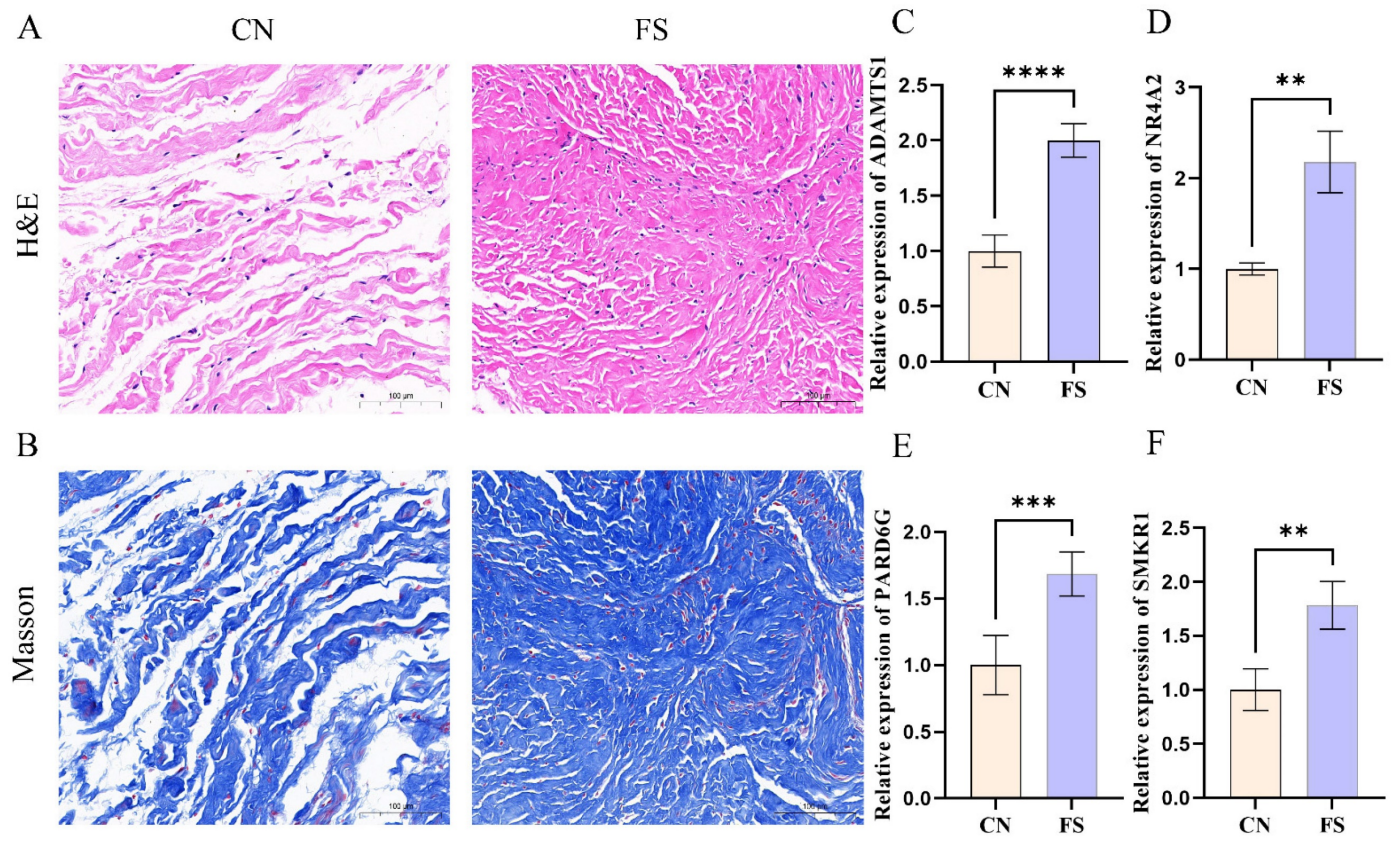
** Aggravated fibrosis and RT-qPCR analysis and in the synovial tissues of FS.** (A) H&E staining in the FS group and control group, scale bar = 100 μm, n = 12 in the FS group; n = 12 in the control group. (B) Masson Trichrome staining in the FS group and control group, scale bar = 100 μm, n = 6 in the FS group; n = 6 in the control group. (C-F) RT-qPCR validated the mRNA expression levels of genes. CN, control; FS, frozen shoulder. **p< 0.01, ***p< 0.005, ****p< 0.001 vs. control group.

To ensure the robustness of our analyses, synovial tissue samples were obtained from individuals diagnosed with FS and rotator cuff tears. Histological examinations, including H&E staining and Masson's trichrome staining, were subsequently performed on these synovial tissue samples. The histological results revealed a significant increase in the number of collagen fibers and fibroblast cells within the synovial tissue of FS patients, with a denser arrangement of collagen fibers observed (Figure 4A, B). Additionally, RT-qPCR was employed to assess the expression levels of key genes. Consistent with the above findings, our results demonstrated up-regulation of ADAMTS1, NR4A2, PARD6G, and SMKR1 in FS samples as compared to control samples (Figure 4C, D, E, F).

### 3.5 Construction of nomogram model for FS risk prediction

**Figure 5:**
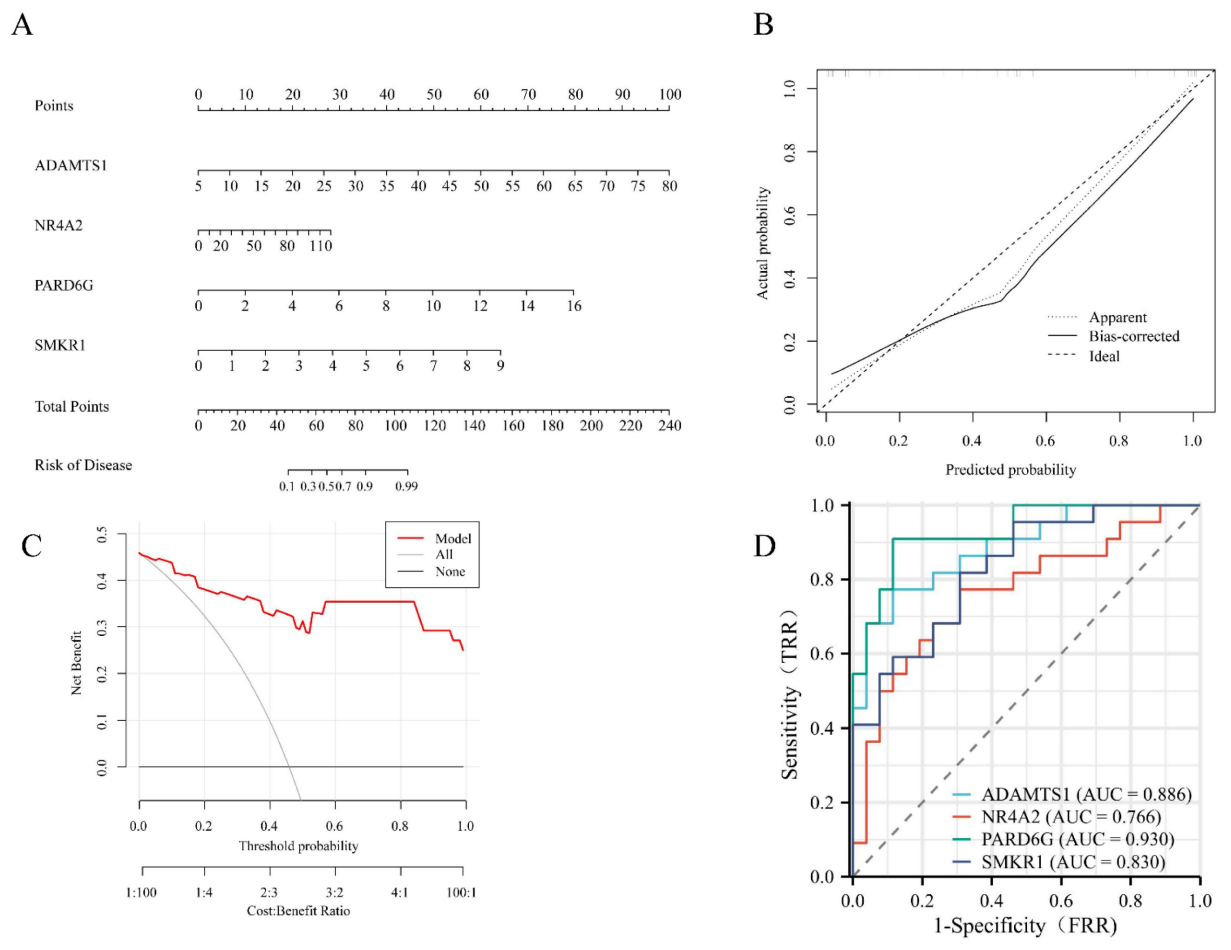
** Development of the diagnostic nomogram model and efficacy assessment.** (A) The nomogram was constructed based on the hub genes. (B) The calibration curve of nomogram model prediction in FS. The dash line is marked as “Ideal”, which represents the standard curve, and is on behalf of the perfect prediction of the ideal model. The dotted line is marked as “Apparent”, which indicates the uncalibrated prediction curve, while the solid line is marked as “Bias-corrected” and represents the calibrated prediction curve. (C) DCA for the nomogram model. The black line is marked as “None”, which stands for the net benefit of the assumption that no patients have FS. The grey line is marked as “All”, which indicates the net benefit of the assumption that all patients have FS, and the red line is marked as “Model” and represents the net benefit of the assumption that FS are identified according to the diagnostic value of predicted by the nomogram model. (D) ROC curves to assess the diagnostic efficacy of each hub gene. ROC receiver operating characteristic, DCA decision curve analysis, FS frozen shoulder.

A nomogram model was constructed to predict the risk of FS. In the column line graph, each trait gene corresponds to a specific point, and the total points are obtained by summing the points of all trait genes. These total points correspond to different FS risk levels (Figure 5A). The calibration curves uncovered that the predicted probability of the constructed nomogram diagnostic model was almost identical to that of the ideal model (Figure 5B). Moreover, the DCA for the nomogram was also performed, showing that decision-making according to the nomogram model may be beneficial for the diagnosis of FS (Figure 5C). Additionally, ROC curves were calculated for the hub genes to evaluate their diagnostic effectiveness. The AUC of our nomogram exhibited good discriminative ability between FS cases and controls. Specifically, the AUC values for ADAMTS1, NR4A2, PARD6G, and SMKR1 were 0.886, 0.766, 0.930, and 0.830, respectively (Figure 5D).

### 3.6 Assessment of immune cell infiltration in FS

**Figure 6:**
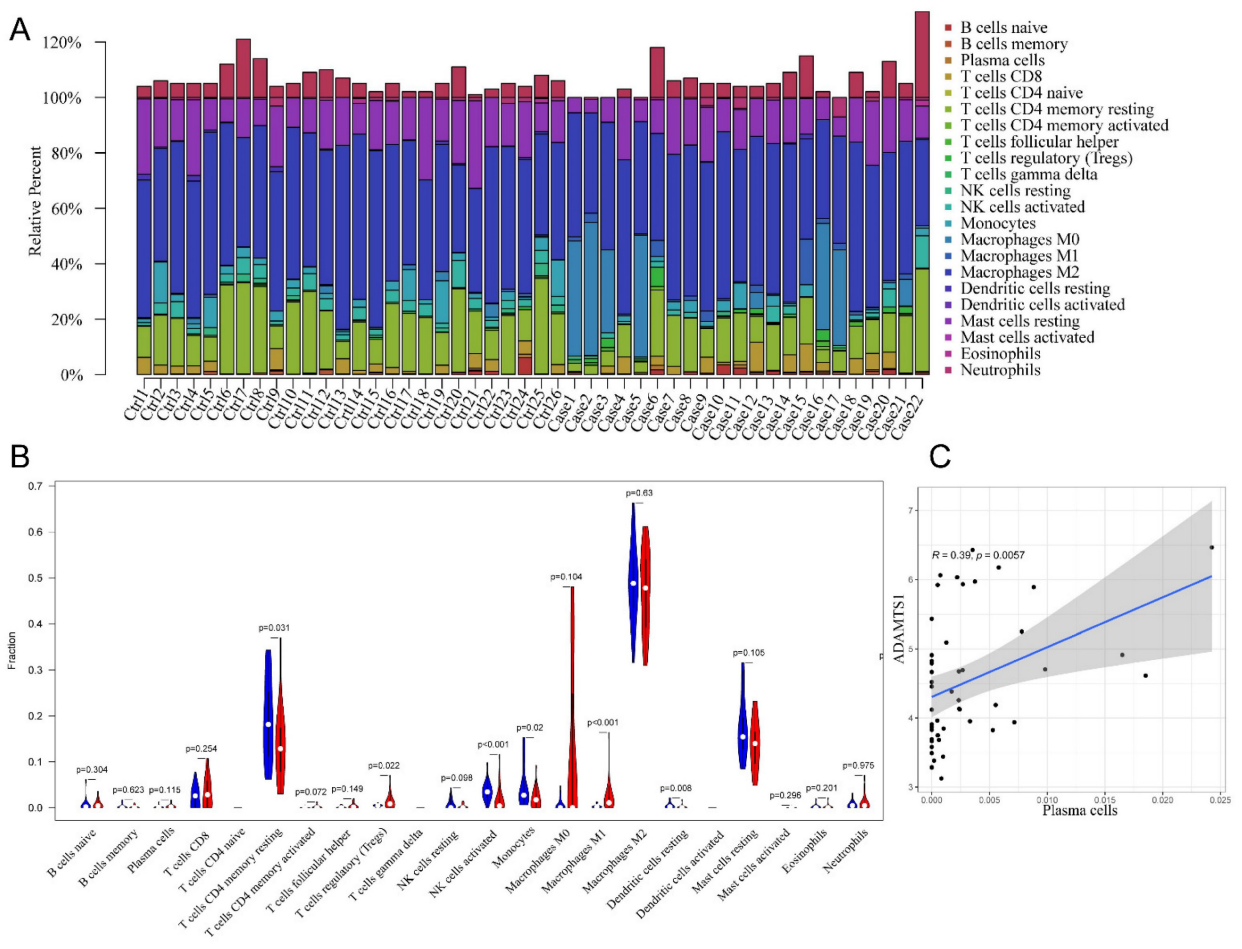
** The results of immune cell infiltration analysis.** (A) The composition of 22 types of infiltrating immune cells in each sample was shown in a bar chart. (B)Wilcoxon test was used to identify significantly different infiltrating immune cells in FS (red) and control (blue) tissues. (C) Significantly correlated hub genes and immune cells were screened by adjusted p-value < 0.05.

In the initial analysis, a bar chart was employed to illustrate the distribution of 22 types of immune cells within each sample (Figure 6A). Each bar in the chart represented the estimated proportion of distinct immune cells present in the sample, with the cumulative proportion summing to 1. Notably, color coding was utilized to denote the relative abundance of different immune cell types across samples. Subsequently, a heatmap was utilized to depict disparities in immune cell abundance between FS samples and control samples. The findings revealed that mast cells in a resting state, macrophages of the M2 subtype, resting memory CD4+ T cells, and naïve B cells constituted the primary infiltrating immune cell populations. Additionally, the violin plot provided insight into the relative infiltration levels of various immune cell subtypes between the FS and control groups (Figure 6B). Specifically, regulatory T cells (Tregs) and M1 macrophages exhibited heightened infiltration levels in the FS group compared to the control group. Conversely, the FS group demonstrated diminished infiltration levels of resting memory CD4+ T cells, activated natural killer cells, monocytes, and resting dendritic cells relative to the control group. Lastly, the results unveiled a significant correlation between plasma cell and ADAMTS1 expression in FS (Figure 6C) (p < 0.05).

## 4. Discussion

FS is a condition characterized by the development of a thickened, fibrotic joint capsule, joint contraction, and reduced intra-articular volume 27. The pathogenesis of FS remains elusive, with its pathological features being multifaceted and including inflammation, pro-inflammatory cytokines, neural and vascular alterations, fibrosis, as well as metabolic and immunological factors 28. Recent research has shed new light on the complex interplay between the brain and the immune system in FS pathology 29, suggesting that central nervous system (CNS) dysfunction may play a significant role in the development and persistence of this condition. Longitudinal studies have explored the clinical course and correlations in FS, highlighting the influence of autonomic function, central pain processing, and psychological variables on patient outcomes 30-32. These findings underscored the multifaceted nature of FS as a "mystery syndrome" 28 and suggested that a comprehensive approach to understanding its pathogenesis and management is necessary.

While adhesive capsulitis is most commonly associated with the shoulder, reports of adhesive capsulitis affecting other joints, such as the ankle 33, demonstrated the potential for shared pathogenic mechanisms across different anatomical sites. Additionally, certain patient populations, such as those with diabetes, may be at increased risk for developing shoulder dysfunction due to a complex interplay of metabolic, immunologic, and neuro-vascular factors 34. Recently, various treatments for FS have been developed, including physiotherapy, pharmacological interventions, corticosteroid injections, and surgical options 1, 35, 36. However, these treatments primarily aim to alleviate symptoms, particularly pain relief and the restoration of mobility and function. In light of the emerging evidence for CNS involvement in FS, there is growing interest in exploring the potential benefits of adding CNS-focused interventions to standard manual therapy and stretching programs 37, 38. As our understanding of the complex pathogenesis of FS continues to evolve, there is a compelling need to explore the underlying mechanisms in greater depth. By leveraging advancements in high-throughput microarray technology and bioinformatics methods, potential core genes that play a significant role in the pathological processes of FS could be identified. Integration of these molecular insights with clinical and neurophysiological findings may pave the way for the development of more targeted and effective diagnostic and therapeutic strategies for this challenging condition.

Diagnostic biomarkers are developed to identify patients with pathological changes. Currently, researchers are focused on validating biomarkers associated with FS. A recent study utilized a machine learning approach to identify central genes in FS, pinpointing MMP9, FOS, SOCS3, and EGF as potential targets 23. In a previous study, Qiao *et al.* identified Myh3 and Srsf1 as key regulatory proteins involved in the development of FS 36. However, to date, there has been no comprehensive validation or assessment of the diagnostic efficacy of these candidate biomarkers using mathematical modeling. MR, as a genetic epidemiological method, can overcome the limitations associated with traditional observational studies. In this study, we utilized the publicly available dataset GSE140731, which is linked to FS, to conduct an innovative combined analysis integrating DEGs and WGCNA. This integrated approach facilitated the identification of co-expressed genes associated with FS and elucidated their functional roles. Subsequently, we innovatively integrated the eQTLs of these co-expressed genes with GWAS data related to FS, leading to the identification and experimental validation of four genes implicated in the onset and progression of FS: ADAMTS1, NR4A2, PARD6G, and SMKR1. Finally, we assessed the diagnostic utility of these four core genes and investigated their association with immune infiltration. To the best of our knowledge, this study was the first to employ such a multifaceted approach in identifying biomarkers for FS.

In this investigation, a comprehensive bioinformatics methodology was employed to identify 295 co-expressed genes by intersecting the screened DEGs with key modules identified through WGCNA. Results from GO and KEGG enrichment analyses revealed that those co-expressed genes primarily participated in biological processes related to extracellular matrix organization, collagen metabolism, and endoplasmic reticulum function. Moreover, KEGG pathway analysis unveiled significant enrichment in pathways including cytokine-cytokine receptor interaction, TNF signaling, and AGE-RAGE signaling in diabetic complications. Noteworthy pathways such as relaxin signaling, ECM-receptor interaction, interleukin-17 (IL-17) signaling, and Toll-like receptor signaling were also prominently enriched. Previous studies by Yang *et al.* and Yano *et al.* had implicated interleukin-6 (IL6) and advanced glycation end-products (AGEs), respectively, in the fibrotic process of FS. Specifically, IL6 had been shown to promote fibrosis via the PI3K-Akt signaling pathway, while AGEs contributed to fibrosis through the activation of NF-kB signaling by binding to RAGE 39, 40. Additionally, relaxin, a peptide hormone, had been identified as capable of inhibiting myofibroblast activation in inflammation and fibrosis by activating multiple signaling pathways 41. Furthermore, research by Moeed *et al.* had indicated that IL-17 produced by T cell subsets within joint capsule tissue induced fibrosis and inflammation of fibroblasts by upregulating its signaling receptor expression 42. Consistent with those findings, previous pathological studies had characterized FS histologically by the presence of type I and type III collagen matrices populated with fibroblasts and myofibroblasts 43-45. Clinically, the symptoms of pain and restricted range of motion observed in patients were largely attributed to chronic inflammation and fibrosis of the joint capsule, aligning with the results of our investigation.

ADAMTS1, a protein with multifaceted biological functions, plays a crucial role in various processes such as extracellular matrix organization, angiogenesis, and inflammation regulation 46, 47. Notably, ADAMTS1 is involved in the regulation of extracellular matrix organization and fibrosis, making it a key player in these mechanisms 48. Studies conducted on mouse cardiac tissue have demonstrated that the cysteine-rich acidic secreted protein leads to age-dependent collagen deposition in the heart by upregulating ADAMTS1 levels 49. Moreover, increased expression of ADAMTS1 in rat kidney tissue has been associated with fibrosis and inflammation 50. In mouse muscle tissue, silencing ADAMTS1 expression has shown promise in alleviating muscle fibrosis 51. In addition to its role in fibrosis, ADAMTS1 has been implicated in the regulation of angiogenesis. Research on mouse kidney tissue has suggested that ADAMTS1 may promote the formation of unstable blood vessels by modulating the signaling mediated by VEGFR2 52. Similarly, in mouse skin tissue, ADAMTS1 has been found to participate in pathological angiogenesis induced by VEGF-A/VPF 53. However, the involvement of the ADAMTS1 gene in FS remains unexplored. Our findings indicate a significant causal relationship between elevated ADAMTS1 expression and the occurrence of FS. We postulate that under pathological conditions, ADAMTS1 promotes the development of FS by inducing local collagen deposition, fibrosis, and angiogenesis.

NR4A2, belonging to the nuclear receptor superfamily, acted as a ligand-activated transcription factor with diverse roles in various organs 54. Studies had indicated the regulatory effects of NR4A2 on pro-inflammatory cytokine expression and fibrosis in different diseases 54, 55. For instance, in a mouse model of multiple sclerosis, abnormal NR4A2 expression enhanced the promoter activity of interleukin-17 and interferon-gamma genes, resulting in excessive cytokine production 56. Similarly, aberrant NR4A2 expression in mouse liver tissue induced inflammation and fibrosis 54. However, there was a scarcity of studies investigating the association between NR4A2 and FS. In our MR analysis, we observed a causal relationship between increased NR4A2 expression and the occurrence of FS. We hypothesized that elevated NR4A2 expression might trigger FS by promoting the expression of inflammatory factors and fibrosis. The PAR6 protein family, initially identified in Caenorhabditis elegans and fruit flies, was known to be essential for establishing cell polarity 57. In mammals, there were three homologs of Par6: PARD6A, PARD6B, and PARD6G. Notably, specific gene knockout studies had suggested that PARD6G was involved in regulating osteoblast proliferation and differentiation, thereby implicating it in cell proliferation 58. However, the functions of PARD6G and SMKR1 genes in the context of FS remained largely unexplored. Further research was needed to elucidate the precise roles of these two genes in the pathogenesis of FS.

FS has been reported to begin as an immune response that exacerbates inflammatory synovitis, subsequently leading to capsular fibrosis 59. Hand *et al.* documented the presence of immune cells, including B-lymphocytes, T-lymphocytes, macrophages, and mast cells in the synovium and capsule of the rotator interval, indicating an immune response in FS 59. However, a comprehensive assessment of immune cell infiltration in FS using CIBERSORT has not been reported. Our results indicate that M1 macrophages and Tregs were highly infiltrated in the synovial tissues of FS patients. During the early stages of wound repair, unstimulated M0 macrophages were converted to M1 macrophages, producing cytokines such as TNF-α and nitric oxide, which amplified the inflammatory response and promoted myofibroblast proliferation and fibroblast recruitment 60. Therefore, we speculated that M1 macrophages played a role in promoting chronic inflammatory responses and accelerating fibrosis in the early stages of FS. Another study reported that Tregs could secrete the pro-fibrotic cytokine transforming growth factor beta 61. Boveda-Ruiz *et al.* found that Tregs played a role in promoting pulmonary fibrosis in a mouse model but exerted a suppressive role in the late stage of the disease 62, Similar complex functions of Tregs during fibrosis progression have been observed in other studies 63. Consequently, we hypothesize that the role of Tregs might be involved throughout the development of FS, exerting either protective or pathogenic effects at different stages of the disease.

Additionally, we investigated the critical role of immune-infiltrating cells and core genes in the context of FS. Our findings revealed a significant positive correlation between ADAMTS1 and plasma cells (r = 0.39, p = 0.0057). Previous research has highlighted the essential function of plasma cells as key effectors in the progression of fibrosis, particularly in mouse lung tissue 60. Therefore, these results provide further evidence supporting the potential influence of ADAMTS1 on fibrosis advancement in FS through the regulation of plasma cell functions. This finding emphasizes the importance of immune-infiltrating cells and their interactions with core genes in the pathogenesis of FS.

However, this study had several limitations that must be acknowledged. Firstly, the GSE140731 dataset utilized in this research was obtained from a public database, and there was a lack of clear definition regarding the staging of FS patients. This ambiguity in patient staging might potentially impact the interpretation of the obtained results. Furthermore, the precise molecular mechanisms underlying the pathogenesis of FS, specifically concerning the upregulation of ADAMTS1, NR4A2, PARD6G and SMKR1, necessitated further investigation through future animal and cell experiments. These additional studies would contribute to a more comprehensive understanding of the role and function of these genes in FS pathology.

## 5. Conclusions

In this study, a comprehensive and innovative approach was undertaken by incorporating a combination of WGCNA, MR analysis, and clinical specimen validation. By employing these advanced methodologies, we effectively identified ADAMTS1, NR4A2, PARD6G, and SMKR1 as potential diagnostic biomarkers for FS. This novel finding not only enhances our understanding of the diagnostic landscape of FS but also offers valuable insights into the underlying molecular mechanisms contributing to its pathogenesis.

## Supplementary Material

Supplementary figure 1, tables 1 and 6.

Supplementary tables 2-5.

## Figures and Tables

**Table 1:** Results of causal effects of 4 co-expressed genes (exposure) on FS (outcome).

Exposure	SNP	Outcome	Methods	OR	SE	95%CI	P value
NR4A2	1	Frozen shoulder	Wald ratio	1.28	0.12	(1.02, 1.61)	0.03
PARD6G	1	Frozen shoulder	Wald ratio	1.27	0.10	(1.04, 1.54)	0.02
SMKR1	1	Frozen shoulder	Wald ratio	1.50	0.15	(1.12, 2.02)	0.01
ADAMTS1	4	Frozen shoulder	IVW	1.12	0.04	(1.02, 1.24)	0.02
			Weighted median	1.15	0.04	(1.06, 1.24)	0.01
			MR Egger	1.22	0.06	(1.09,1.36)	0.07

Abbreviations: SNPs, single nucleotide polymorphisms; OR, odds ratio; SE, standard error; CI, confidence interval. NR4A2, nuclear receptor subfamily 4 group A member 2; PARD6G, Par-6 family cell polarity regulator Gamma; SMKR1, small lysine rich protein 1; ADAMTS1, ADAM metallopeptidase with thrombospondin type 1 motif 1; IVW, inverse variance weighting.
