# Supplementary material for: Exploring Pathogenic Genes in Frozen Shoulder through weighted gene co-expression network analysis and Mendelian Randomization
Source: Int J Med Sci. 2024 Oct 21;21(14):2745–58. doi: 10.7150/ijms.98505 (PMC11539380; doi:10.7150/ijms.98505)
Supplement: Supplementary file 1 — Supplementary figure 1, tables 1 and 6. [file ijmsv21p2745s1.pdf]

Supplementary Figure 1

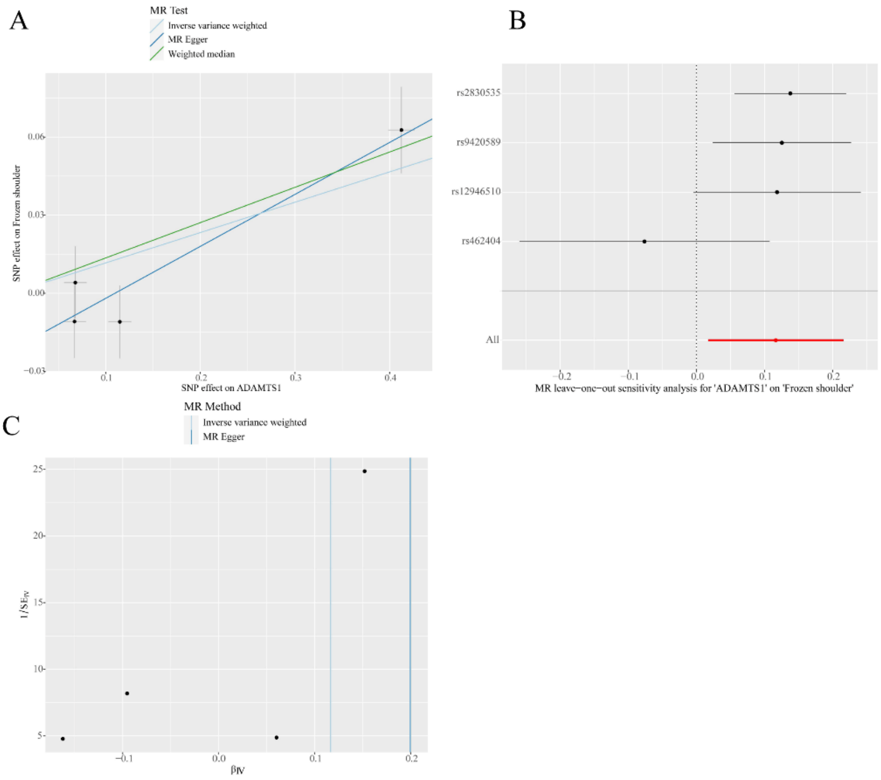

Supplementary Figure 1. MR results of ADAMTS1 on frozen shoulder. (A) Scatter plot showing the causal effect of ADAMTS1 on the risk of FS. (B) Leave-one-out plot to visualize the causal effect of ADAMTS1 on FS risk when leaving one SNP out. (C) Funnel plots to visualize the overall heterogeneity of MR estimates for the effect of ADAMTS1 on FS.

Supplementary Table 1

Patient information

sample	Sex	Age (years)	abduction(passive)	external rotation(passive)	flexion(passive)
FS1	Female	63	82	21	68
FS2	Female	69	61	16	52
FS3	Female	64	72	22	92
FS4	Male	43	64	15	93
FS5	Female	57	63	16	85
FS6	Male	62	77	19	70
FS7	Female	64	64	14	86
FS8	Female	54	56	16	62

FS9	Male	59	45	17	63
FS10	Female	48	60	13	90
FS11	Male	62	62	15	78
FS12	Female	53	55	17	93
CN1	Female	74	147	60	162
CN2	Female	74	130	64	157
CN3	Male	41	157	58	160
CN4	Female	57	160	64	173
CN5	Female	59	163	65	170
CN6	Female	53	143	53	161
CN7	Female	64	140	60	163
CN8	Male	56	150	57	153
CN9	Male	43	162	59	158
CN10	Female	69	170	65	177
CN11	Female	53	130	58	154
CN12	Female	65	140	64	163

Supplementary Table 6

Heterogeneity test of ADAMTS1 on frozen shoulder

exposure	outcome	method	Q	Q_df	Q_pval
ADAMTS1	Frozen shoulder	MR Egger	1.57708	2	0.454508
ADAMTS1	Frozen shoulder	Inverse variance weighted	5.639798	3	0.130512

Pleiotropy test of ADAMTS1 on frozen shoulder

exposure	outcome	method	egger_intercept	pval
ADAMTS1	Frozen shoulder	MR Egger	-0.02192	0.181394
